# Fully Automated Segmentation of Lower Extremity Deep Vein Thrombosis Using Convolutional Neural Network

**DOI:** 10.1155/2019/3401683

**Published:** 2019-06-09

**Authors:** Chen Huang, Junru Tian, Chenglang Yuan, Ping Zeng, Xueping He, Hanwei Chen, Yi Huang, Bingsheng Huang

**Affiliations:** ^1^Department of Radiology, Guangzhou Panyu Central Hospital, Guangzhou, China; ^2^Medical Imaging Institute of Panyu, Guangzhou, China; ^3^School of Biomedical Engineering, Health Science Center, Shenzhen University, Shenzhen, China; ^4^Shenzhen University Clinical Research Center for Neurological Diseases, Shenzhen, China

## Abstract

**Objective:**

Deep vein thrombosis (DVT) is a disease caused by abnormal blood clots in deep veins. Accurate segmentation of DVT is important to facilitate the diagnosis and treatment. In the current study, we proposed a fully automatic method of DVT delineation based on deep learning (DL) and contrast enhanced magnetic resonance imaging (CE-MRI) images.

**Methods:**

58 patients (25 males; 28~96 years old) with newly diagnosed lower extremity DVT were recruited. CE-MRI was acquired on a 1.5 T system. The ground truth (GT) of DVT lesions was manually contoured. A DL network with an encoder-decoder architecture was designed for DVT segmentation. 8-Fold cross-validation strategy was applied for training and testing. Dice similarity coefficient (DSC) was adopted to evaluate the network's performance.

**Results:**

It took about 1.5s for our CNN model to perform the segmentation task in a slice of MRI image. The mean DSC of 58 patients was 0.74± 0.17 and the median DSC was 0.79. Compared with other DL models, our CNN model achieved better performance in DVT segmentation (0.74± 0.17 versus 0.66±0.15, 0.55±0.20, and 0.57±0.22).

**Conclusion:**

Our proposed DL method was effective and fast for fully automatic segmentation of lower extremity DVT.

## 1. Introduction

Deep vein thrombosis (DVT) is a disease caused by abnormal blood clots in deep veins, which generally occurs in the lower extremity [1]. As a common peripheral vascular disease, DVT has annual incidence of about 0.1%, which is increasing year by year [2]. DVT often results in complications such as pulmonary embolism (PE) and postthrombotic syndrome (PTS), which significantly affect the quality of life of patients [2] and even cause death [3].

The lower extremity DVT mainly includes distal DVT and proximal DVT, which occurs above the knee and below the knee, respectively [4]. In general, the lesion's extent and volume of distal DVT is smaller than that of proximal DVT. The clinical diagnosis of lower extremity DVT is unreliable due to the poor specificity of signs and symptoms [5–7]. Imaging examination is more objective and definitive than clinical diagnosis [8]. Accurate and timely diagnosis is of great significance to the treatment and prognosis of lower extremity DVT patients [9].

Since magnetic resonance imaging (MRI) is noninvasive and is not associated with radiation exposure, it has become a useful imaging modality in thrombus diagnosis [10]. In contrast-enhanced MRI (CE-MRI), thrombus can be better detected as a contrast-filling defect. CE-MRI has been suggested as a standardized imaging examination for DVT in some studies [11]. Accurate segmentation of thrombotic lesions by CE-MRI is important to determine the extent and volume of thrombus, which can facilitate the diagnosis and treatment.

Manual delineation of the thrombus volumes in CE-MRI is time-consuming and laborious. In addition, manual delineation is subjective and the result depends on the experience of clinicians. Automatic segmentation can be faster and reproducible compared to manual delineation. Semiautomatic segmentation could take advantage of the prior knowledge of clinicians but still remains time-consuming. In fully automatic segmentation, thrombus with various irregular shape would increase the complexity of segmentation. The tissues with similar intensity as thrombus are likely to be identified as thrombus. To date, no studies about automatic segmentation of DVT have been reported. Some automatic segmentation methods have been applied to lower limb blood vessel segmentation [12, 13], such as fuzzy connected object delineation algorithms [14], k-means [15], fuzzy c-means clustering [16], and convolutional neural network (CNN) [17]. Among these automated methods, machine learning (ML), especially deep learning (DL), has shown great potential [18].

In the current study, we adopted a fully automatic method of DVT delineation based on DL and CE-MRI images, aiming to reduce the burden of clinicians and to improve the efficiency and accuracy of DVT segmentation.

## 2. Materials and Methods

### 2.1. CE-MRI Images

From November 2016 to January 2018, CE-MRI images of 58 patients with newly diagnosed lower extremity DVT were recruited from Guangzhou Panyu Central Hospital, including 25 males and 33 females aged between 28 and 96 years. Gadopentetate Dimeglumine was injected intravenously before MRI scanning. The CE-MRI images were acquired in the lower extremity on a 1.5 T system (Avanto, Siemens, Germany). Protocol parameters were as follows: repetition time = 3.17 ms, echo time = 1.13 ms, field of view = 500 ×500 mm^2^, flip angle = 25°, and spatial resolution =0.75×0.55×0.6 mm^3^. The image matrix of most images was 961×345. For each patient, the CE-MRI images with lower extremity DVT were collected in three stages, and each stage's scanning time was about 33 seconds. There were a total of 5388 slices of CE-MRI acquired for 58 patients, 2683 of which had thrombus lesions, accounting for around 50% of the total slices.

### 2.2. Data Preprocessing

The ground truth (GT) of thrombus lesions was manually contoured with the consensus between two experienced radiologists on the CE-MRI using the ITK-SNAP software (http://www.itksnap.org) [19]. To speed up gradient descent and search for the optimal solution, the original data were normalized by performing min-max normalization as follows:

(1) $$\begin{array}{c} X*=\frac{X-X_{m}}{X_{M}-X_{m}} \end{array}$$

where X denotes images and X_m_ and X_M_ denote the minimum and maximum gray value of X, respectively. X*∗* denotes the result of normalization. In order to fit our CNN network, all MRI images were resized to 960×320 with an embedded Matlab function (Imresize subroutine of Matlab, Natick, MA, USA). Since DL method demands huge amount of input data in training, we augmented our training dataset by rotating each slice between -2 degrees and 0 degrees with an interval of 2 degrees, scaling between 0.9 multiples and 1.1 multiples with an interval of 0.1 multiples and horizontal mirroring for each slice. Ultimately, we acquired a total of 63126 (5388×12) slices of images.

### 2.3. CNN Network

**Figure 1:**
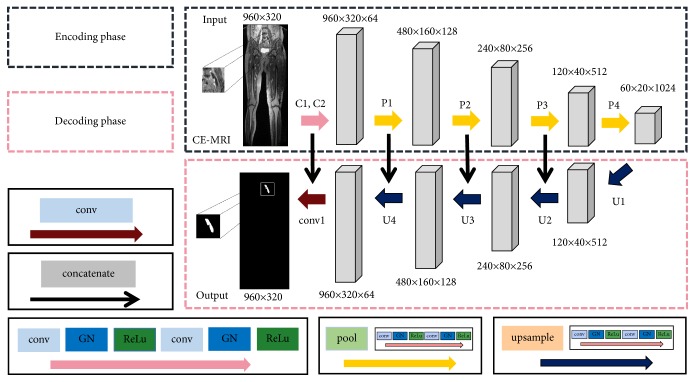
The architecture of the proposed convolutional neural network (CNN) model. The proposed CNN network includes two phases: encoding phase and decoding phase. The encoding phase consists of 2 Conv-Group normalization (GN)—ReLu blocks (C1-C2) and 4 pooling blocks (P1-P4). The decoding phase consists of 4 upsampling blocks (U1-U4) and one convolution layer (conv1). The output of each layer is a three-dimensional matrix with the size of h×w×d, where h and w are the length and width of the feature map, respectively, and d is the feature dimension.

Our CNN network with an encoder-decoder architecture designed for DVT segmentation was inspired by fully convolutional network [20] and U-Net [21], as shown in Figure 1. The purpose of the encoding phase was to extract the feature information of MRI images and represent the high-level features with semantic information. The encoding phase (C1-C2, P1-P4) includes 2 Conv-Group normalization (GN) [22]—ReLu blocks (C1-C2) and 4 pooling blocks (P1-P4). The Conv-GN-ReLu block consists of one convolution layer, one GN layer, and one ReLu layer, while the pooling block consists of one pooling layer (Pool) and 2 Conv-GN-ReLu blocks. The convolution layer detects abstract features and semantic information from the input images, and the ReLu layer accelerates the training and convergence of our network model. In the pooling layer (P1-P4), max-pooling with 2 × 2 filters was designed to decrease the computational time and connection parameters. The GN layer is a new normalization method, which could accelerate convergence and improve network performance more stably [22]. The feature map size is reduced from 960×320 to 60×20 when the encoding phase is finalized.

Decoding phase (U1-U4, conv1) with upsampling blocks could reconstruct the feature maps from 60 × 20 in the encoding phase to 960 × 320. There are 4 upsampling blocks (U1-U4) and one convolution layer (conv1) in the decoding phase. An upsampling block consists of one upsampling layer, one concatenate layer, and 2 Conv-GN-ReLu blocks. Since some details of the images could be lost and the resolution of images might be reduced in the reconstruction of feature images, concatenate layers are designed for feature fusion of high-resolution and low-resolution feature maps. When all the upsampling blocks are finalized, the feature maps are reconstructed to an output image with the same size of 960×320, as the original input MRI images. To optimize the network, we used focal loss [23] as the loss function, which was modified on the basis of standard cross-entropy loss.

### 2.4. Network Training and Testing

58 patients were randomly divided into 8 groups for 8-fold cross-validation strategy. For each cross-validation, seven groups of patients were used as training sets and the remaining group was testing set.

During training, the parameters were set as follows: basic learning rate, 1×10^−3^; batch size, 2; gamma, 0.1; and momentum, 0.9. Optimization was performed by using Adam [24]. Based on the Keras framework (http://keras.io/), our model used a GPU NVIDIA GeForce GTX 1080TI equipped on an Intel Xeon E5-2650 2.30GHz×12 machine with Linux Ubuntu 14.04 operating system. Our CNN model had about 7.8 million parameters in total. It took about 48 hours (250 epochs) for the whole training.

After adequate training on the network, testing set was used to test the network's performance. Metrics of dice similarity coefficient (DSC), precision, and recall [25, 26] were adopted to evaluate the difference between segmentation results and GT in current study. All of these measurements range from 0 to 1, indicating the inferior to the superior performance of the segmentation algorithm. The formulae of these measurements are as follows:

(2) DSC=2TPFP+2TP+FN

(3) precision=TPTP+FP

(4) recall=TPTP+FN

True positive (TP) denotes the number of pixels of DVT lesion area which are correctly identified, false positive (FP) denotes the number of pixels of normal tissue which are wrongly recognized as the lesion, and false negative (FN) denotes the number of pixels of the lesion area which are wrongly predicted as normal tissue. DSC describes the overlap between the GT and the automatic segmentation result. Precision shows the proportion of correctly identified lesion area in all the identified “lesion areas.” Recall means the proportion of correctly identified lesion area in the ground truth.

### 2.5. Comparison with Other Models

Since no studies about automatic segmentation DVT lesions are available in literature, we tried to apply some classic segmentation methods on our dataset for comparisons with our proposed method, such as original U-Net [21], Segnet [27], and Global Convolutional Network (GCN) [28]. The preprocessing, loss function, and training strategy (8-fold cross-validation) were the same as in the present work. DSC, precision, and recall were also computed. Statistical significance of the observed differences was determined using the two-sided paired Wilcoxon signed-rank test, and p value < 0.05 was considered significant.

## 3. Results

**Figure 2:**
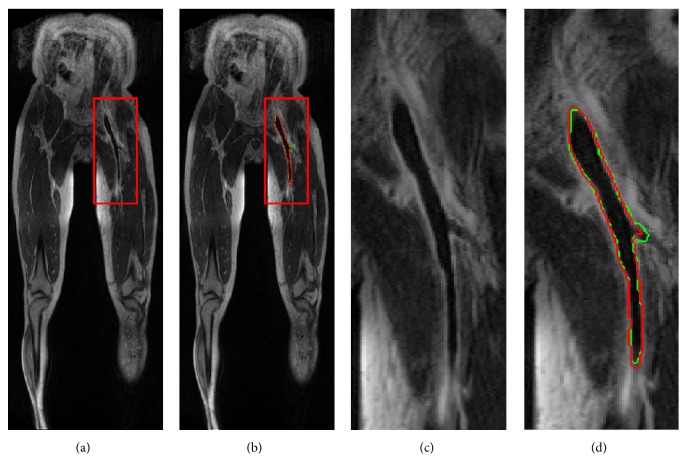
An example of DVT segmentation with high accuracy. The dice similarity coefficient (DSC) was 0.94. (a) CE-MRI image. (b) Automatic segmentation (red line) and ground truth (GT) (green line) presented on CE-MRI image. (c) Magnification of the red box area in (a). (d) Magnification of the red box area in (b).

It took about 1.5s for our CNN model to perform the segmentation task in a slice of MRI image. The segmentation task for a DVT patient with around 90 slices of MRI images required around 13.5 seconds. The mean DSC of 58 patients was 0.74± 0.17 and the median DSC was 0.79 (range, 0 ~0.91). A typical example of thrombus segmentation with high accuracy was shown in Figure 2, in which the DSC was 0.92. The different segmentation performances of proximal DVT and distal DVT are presented in Table 1. Compared with that of the distal DVT, the mean DSC of proximal DVT was higher (0.78±0.12* versus *0.57±0.19).

**Figure 3:**
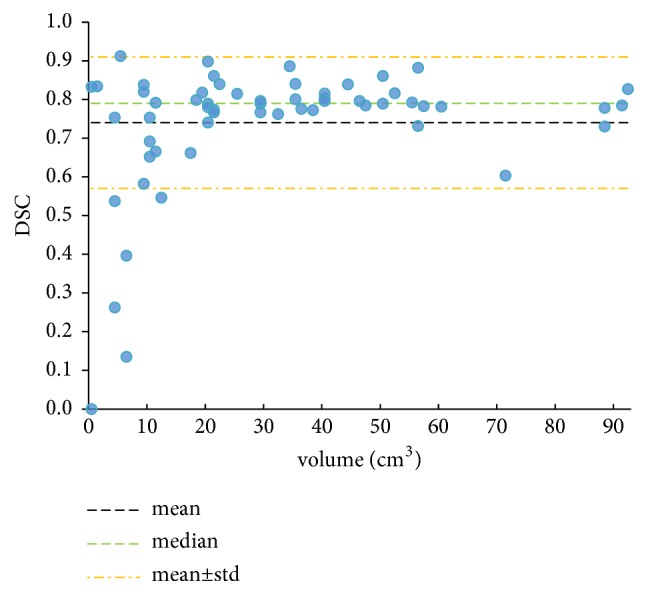
The thrombus volumes and DSCs of all patients. Each point represents a patient.

The thrombus volumes and DSCs for all patients are shown in Figure 3. In most patients, CNN model resulted in high DSC, in two patients even higher than 0.90. In 7 patients, the segmentation was unsatisfactory, with DSC below 0.60.

Table 2 shows the comparison of segmentation performance in terms of DSC, precision, and recall between our CNN network and other models. The mean DSC of original U-Net, Segnet, and GCN were 0.66±0.15, 0.55±0.20, and 0.57±0.22, respectively. Our proposed method has achieved significant advantages in comparison with the other three models (all p < 0.001).

## 4. Discussion

In the current study, we proposed a fully automatic segmentation method based on DL and CE-MRI images for DVT lesion segmentation. Our method achieved good performance with mean DSC of 0.74 and median DSC of 0.79 (range, 0~0.91), indicating the great potential of deep learning in DVT lesion detection and segmentation.

As shown in Table 2, our proposed method has achieved better performance in DVT segmentation than other classic models. Compared with original U-Net, we added GN layers in our proposed model and this modification made the network more effective. The large convolution kernel size with the large receptive field in GCN may not be suitable for the current task, since the thrombus volume is generally small. In Segnet, five pooling layers may be too many to extract suitable features of small thrombus for successful detection and segmentation.

Such good performance may be firstly attributed to the modified U-Net architecture in our study, especially with such limited computational resources we used. Based on U-Net network, the GN layer was added in our network, which was the only significant modification of U-Net in our study. As known, batch normalization (BN) is a common normalization method in deep learning, which plays an important role in improving training and convergence speed. BN normalization is achieved along the batch dimension and relies heavily on batch size, with larger batch size achieving better normalization effect. However, large batch size requires very large memory consumption and computational resources, while small batch size may cause inaccurate estimation of the batch statistics and thus decrease the accuracy of segmentation [22]. On the contrary, GN was independent of the batch size as it divides the channels of images into groups to calculate the normalized mean and variance [22]. Therefore, the performance of BN may not be satisfactory with small batch size, while the performance of GN was almost unaffected by the limited computational resources and small batch sizes in our study.

The second reason for the good performance may be the focal loss function adopted in our study. Usually, the sizes of DVT lesions were relatively small in CE-MRI images, which makes it difficult for our DL network to fully learn the useful information of lesions during training procedure. Besides, the nonlesion region in CE-MRI images, which is the main part in CE-MRI images, normally contributed significantly to the loss, which dominated the direction of updating the gradient and concealed useful information of the DVT lesions. The focal loss function adopted in our study could solve the challenge of serious imbalance between such nonlesion background and DVT lesions. By using the foal loss function, the weight of the nonlesion background in the training process can be reduced and the DVT lesion can contribute more to the loss. Hence, the focal loss function we selected could make the model focus more on the DVT lesion images and converge faster.

**Figure 4:**
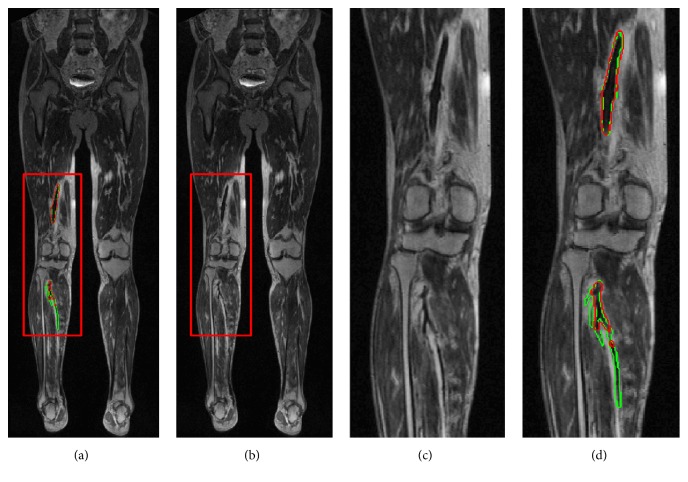
An example of DVT segmentation with both proximal and distal DVTs. The DSC of whole image was 0.78, with 0.92 for proximal DVT and 0.57 for distal DVT. (a) CE-MRI image. (b) Automatic segmentation (red line) and GT (green line) presented on CE-MRI image. (c) Magnification of the red box area in (a). (d) Magnification of the red box area in (b).

The segmentation results of distal and proximal DVT were specifically evaluated in our study. As shown in Table 1, the proximal DVT segmentation results were better than those of distal DVT. A typical segmentation example of both proximal and distal DVT is shown in Figure 4, in which the DSC of proximal DVT was 0.92, while the DSC of distal DVT was only 0.57. The segmentation was unsatisfied in four patients with DSCs below 0.50 (Figure 3), and we found that all their thrombus lesions were in the calf. Distal DVT in narrow calf vein is often difficult to detect because of its complicated anatomy and frequent vascular variation. There have been studies reporting that the sensitivity and specificity of MRI for proximal DVT both exceeded 90%, and pooled sensitivity for distal DVT was about 60% [29]. These may explain the relatively low DSCs in some patients especially with distal DVT.

## 5. Limitations and Future Works

There may be some limitations in the current study. Firstly, more data should be collected to construct a more robust model, and data from multicenters could further verify the generalization ability of our model. Secondly, multisequence information of MRI, such as T2-weighted images, which are also widely used in the diagnosis of DVT, may be incorporated in our model to improve the performance of our method. Finally, in our present study, only 2D images were used to complete the automatic segmentation task and the volumetric information was abandoned. In future work, optimization of the network structure and more computational resource are needed for direct training of the 3D images to achieve better segmentation performance.

## 6. Conclusion

Our study adopted a CNN model to delineate the lower extremity DVT automatically in CE-MRI images. The results showed that our proposed method was relatively effective and fast. If further improved, our method would be helpful in assisting clinicians in rapid and objective evaluation of DVT.

## Figures and Tables

**Table 1:** The segmentation performance of proximal DVT, distal DVT lesions, and all lesions.

		DSC	Precision	Recall
*Proximal DVT*	Mean±std	0.78±0.12	0.75±0.14	0.83±0.12
(56 lesions)	Median	0.81	0.79	0.86
	Range	0.27~0.92	0.16~0.91	0.48~0.99

*Distal DVT*	Mean±std	0.57±0.19	0.64±0.22	0.56±0.24
(43 lesions)	Median	0.62	0.70	0.54
	Range	0~0.86	0~0.90	0~0.99

*All 58 lesions*	Mean±std	0.74±0.17	0.75±0.16	0.72±0.15
	Median	0.79	0.77	0.82
	Range	0~0.92	0~0.88	0~0.99

**Table 2:** Comparisons of segmentation performance between the proposed CNN model and the other models.

Algorithm	*DSC*		*Precision*		*Recall*	
	Mean ± SD	Range	Mean ± SD	Range	Mean ± SD	Range
Original U-Net [21]	0.66±0.15 (p < 0.001)	0~0.84	0.71±0.17	0.05~0.88	0.65±0.17	0.04~0.92
Segnet [27]	0.55±0.20 (p < 0.001 )	0.01~0.81	0.64±0.25	0.01~0.93	0.51±0.19	0.02~0.89
GCN [28]	0.57±0.22 (p < 0.001 )	0.02~0.83	0.77±0.28	0~0.92	0.48±0.22	0.02~0.78
*Proposed method*	*0.74±0.17*	0~0.92	*0.75±0.16*	0~0.88	*0.72±0.15*	0~0.99

Notes: DSC, dice similarity coefficient. The p values are obtained by using two-sided paired Wilcoxon signed-rank tests.

## Data Availability

The authors do not have permission to share data.
